# Prognostic implications of necroptosis-related long noncoding RNA signatures in muscle-invasive bladder cancer

**DOI:** 10.3389/fgene.2022.1036098

**Published:** 2022-12-02

**Authors:** Kan Jiang, Lingyun Wu, Xin Yin, Qiuying Tang, Jie Yin, Ziyang Zhou, Hao Yu, Senxiang Yan

**Affiliations:** ^1^ Department of Radiation Oncology, The First Affiliated Hospital, Zhejiang University School of Medicine, Hangzhou, China; ^2^ Zhejiang University Cancer Center, Hangzhou, Zhejiang, China

**Keywords:** necroptosis, lncRNAs, muscle-invasive bladder cancer, prognosis, immune microenvironment, drug resistance

## Abstract

**Background:** Bladder cancer (BLCA) is the sixth most common cancer in men, with an increasing incidence of morbidity and mortality. Necroptosis is a type of programmed cell death and plays a critical role in the biological processes of bladder cancer (BLCA). However, current studies focusing on long noncoding RNA (lncRNA) and necroptosis in cancer are limited, and there is no research about necroptosis-related lncRNAs (NRLs) in BLCA.

**Methods:** We obtained the RNA-seq data and corresponding clinical information of BLCA from The Cancer Genome Atlas (TCGA) database. The seven determined prognostic NLRs were analyzed by several methods and verified by RT-qPCR. Then, a risk signature was established based on the aforementioned prognostic NLRs. To identify it, we evaluated its prognostic value by Kaplan–Meier (K-M) survival curve and receiver operating characteristics (ROC) curve analysis. Moreover, the relationships between risk signature and clinical features, functional enrichment, immune landscape, and drug resistance were explored as well.

**Results:** We constructed a signature based on seven defined NLRs (HMGA2-AS1, LINC02489, ETV7-AS1, EMSLR, AC005954.1, STAG3L5P-PVRIG2P-PILRB, and LINC02178). Patients in the low-risk cohort had longer survival times than those in the high-risk cohort, and the area under the ROC curve (AUC) value of risk signature was higher than other clinical variables. Functional analyses, the infiltrating level of immune cells and functions, ESTIMATE score, and immune checkpoint analysis all indicated that the high-risk group was in a relatively immune-activated state. In terms of treatments, patients in the high-risk group were more sensitive to immunotherapy, especially anti-PD1/PD-L1 immunotherapy and conventional chemotherapy.

**Conclusion:** The novel NLR signature acts as an invaluable tool for predicting prognosis, immune microenvironment, and drug resistance in muscle-invasive bladder cancer (MIBC) patients.

## Introduction

Bladder cancer (BLCA) is the sixth most common cancer in men, with an increasing incidence of morbidity and mortality. The most common malignant tumor of the urinary system, more than 570,000 patients were diagnosed with BLCA in 2021 (Sung et al., 2021; Siegel et al., 2022). BLCA is usually divided into non-muscle-invasive bladder cancer (NMIBC) and muscle-invasive bladder cancer (MIBC) according to the depth of tumor invasion. Approximately 75% of patients have NMIBC, while roughly 25% of patients have MIBC on account of invasion beyond the muscularis propria (Witjes et al., 2021). Unfortunately, a quarter of NMIBC patients with high-risk features will eventually relapse and evolve into MIBC patients (Babjuk et al., 2019). Although intensive treatment of neoadjuvant chemotherapy and immunotherapy combined with radical cystectomy is effective for MIBC, the 5-year overall survival (OS) rate of MIBC is still less than 50% (Hermans et al., 2018). To overcome it, many efforts had been made for the treatment of MIBC, but there has been no well-accepted therapeutic biomarker to prolong the survival time apart from some immune checkpoints. In addition, it is also crucial to determine which therapeutic strategies can benefit patients, such as immunotherapy. Hence, there is evident clinical significance in identification and validation of novel biomarkers to predict prognosis and therapeutic response of MIBC patients.

Necroptosis is a form of regulated necrotic cell death regulated by receptor-interacting protein kinase 1 (RIPK1), RIPK3, and performed by mixed lineage kinase domain-like pseudokinase (MLKL) (Holler et al., 2000; Cho et al., 2009; Christofferson and Yuan, 2010). It was discovered that necroptosis bears a mechanistic resemblance to apoptosis and a morphological similarity to necrosis. Necroptosis is characterized by early loss of plasma membrane integrity, leakage of intracellular contents, and organelle swelling (Gong et al., 2019). Increasing evidence suggested that necroptosis plays a critical role in multiple cancer biological processes, including pathogenesis, cancer metastasis, cancer immunity, and treatment resistance (Su et al., 2015; Negroni et al., 2020; Sprooten et al., 2020; Park et al., 2021). Some researchers also identified the indispensable role of necroptosis in BLCA. ABT-737, a Bcl-2 inhibitor, can directly induce necroptosis by upregulating RIPK3 in BLCA (Cheng et al., 2021). It was also reported that inhibition of CK1δ activity can trigger necroptosis in BLCA cells, which can be proposed as a novel strategy for antitumor treatment (Lin et al., 2020). Referring to the treatments, a study found that inducing necroptosis was an alternative approach to overcome cisplatin resistance in BLCA therapy (Wang et al., 2018). Unfortunately, only few research studies were carried out on necroptosis in BLCA.

Long noncoding RNAs (lncRNAs), a type of RNA that is more than 200 nucleotides in length, mostly cannot code for proteins (Bhan et al., 2017). Over the last decades, accumulated evidence revealed that lncRNAs were involved in various biological functions and disease processes, including cancers. It is well-recognized that lncRNAs regulate biological mechanisms such as proliferation, energy metabolism, cancer metastasis, immune escape, and drug resistance in BLCA (Robertson et al., 2017; He et al., 2020; Chen et al., 2021a; Tang et al., 2022; Wu et al., 2022). For instance, lncRNA BLACAT2 interacted with WDR5 directly, inducing intratumoral/peritumoral lymphangiogenesis and invasion of BLCA (He et al., 2018). LncRNA LNMAT1 can modulate the tumor microenvironment (TME) in lymphatic metastasis of BLCA by upregulating the expression of CCL2 and recruiting macrophages into the tumor (Chen et al., 2018). Moreover, some lncRNAs possess good capacity of prognostic value. Decreased expression of lncRNA MIR31HG may inhibit cell proliferation and migration and was associated with better OS and disease-free survival (DFS) in MIBC (Wu et al., 2020). However, current studies focusing on lncRNA and necroptosis in cancer are limited, and there are few research studies about necroptosis-related lncRNAs (NRLs) in BLCA. Consequently, it is vital for us to identify key NRLs which can predict the therapeutic response and prognosis of MIBC patients.

In this study, we obtained different NRLs in MIBC from The Cancer Genome Atlas (TCGA) database (http://portal.gdc.cancer.gov/). Then, we classified the MIBC patients and constructed a novel signature based on significant NRLs. Furthermore, the value of this model in predicting prognosis, immune microenvironment, chemotherapy, and immunotherapy response was estimated as well.

## Materials and methods

### Data acquisition and processing

The processed fragments per kilobase of transcript per million mapped reads (FPKM)-standardized RNA-seq data and corresponding clinical information for the BLCA were extracted from the TCGA website. As a result, a total of 411 BLCA samples and 19 normal samples were considered in our study. We first converted the ensemble gene id into gene symbol using Strawberry Perl. During this process, we averaged expression levels of the same gene in multiple lines and filtered through genes that were not expressed in all samples. Then, we obtained the clinical data by excluding patients with unknown survival times. As the targets of our study were MIBC patients, four patients with T1 stage who were deemed to be NMIBC were excluded.

### Identification of prognostic NLRs

Based on previous studies (Zhao et al., 2021), 67 genes were examined to be associated with necroptosis. We acquired the correlation between necroptosis-related genes (NRGs) and lncRNAs through the “limma” R package. As a result, 1,139 lncRNAs, with correlation coefficient > 0.4 and *p* < 0.001, were obtained as NLRs. The “limma” R package was also applied to screen out differentially expressed NRGs and NLRs between tumor and normal samples. (log2fold change (FC) > 1, false discovery rate (FDR) < 0.05, and *p* < 0.05). After collating the overall survival (OS) of each sample, univariate Cox proportional hazard regression analysis was used to analyze NLRs related to the prognosis of MIBC patients (*p* < 0.01). Then, we screened out the candidate NLRs through the least absolute shrinkage and selection operator (LASSO) analysis to avoid overfitting. Finally, the determined prognostic NLRs were obtained by stepwise multivariate Cox proportional hazard regression analysis.

### Cell lines and RT-qPCR analysis

The cells in our study, including normal human bladder epithelial cell lines (SV-HUC1) and MIBC cell lines (T24, TSSCUP, UMUC-3, and EJ), were acquired from the American Type Culture Collection (ATCC; Manassas, VA, United States). The cells were cultured in RPMI 1640 medium (BD) supplemented with 10% FBS and 100 IU/ml penicillin–streptomycin solution at 37°C in 5% CO_2_. Total RNAs were extracted from the cells using a Trizol Kit (Invitrogen, Carlsbad, CA, United States). cDNA was synthesized by reverse transcription using qPCR RT Master Mix (Takara, Japan). The relative expression levels of lncRNAs were determined by the △Ct method using the SYBR Green qPCR Kit (Takara, Japan). Primer sequences are given in Supplementary Table S1.

### Consensus clustering

For exploring potential molecular subtypes in MIBC patients, the “ConsensusClusterPlus” R package was used to sort out the optimal cluster value based on determined prognostic NLRs. After that, we used the “Rtsne” R package to accomplish principal component analysis (PCA).

### Establishment of the necroptosis-related risk model

We established the risk model based on the aforementioned prognostic NLRs, and the computational formula of the risk model is as follows:

$$risk\;score=\overset{n}{\underset{i=1}{\sum }}\left(Coef_{i}*exp_{i}\right.).$$

Here, *Coef* represents the coefficient value, and *exp* represents the expression level of the corresponding NLR. In addition, the Sankey diagram, which visualized the relationship between NRGs and lncRNAs, was constructed using Cytoscape and the “ggalluvial” R package.

### Evaluation of prognostic value of the risk model

We divided all MIBC patients from the TCGA cohort into high-risk and low-risk subgroups according to the median risk score. The Kaplan–Meier method of “survival” R package was carried out to evaluate OS between the two subgroups. To further identify whether the risk model was an independent factor of prognosis, we developed univariate Cox and multivariate Cox proportional hazard regression analyses to affirm it. At the same time, time-dependent receiver operating characteristics (ROC) curves were carried out to compare the different prognostic values of these variables. We also used the chi-square test to analyze the correlation between the risk model and clinical features.

### Construction of nomogram

The clinical features including age, stage, gender, grade, and risk model were applied to the construction of a nomogram for the 1-, 2-, and 3-year OS through the “rms” R package, and we also used a calibration curve to illustrate whether the prediction was consistent with practice.

### Functional and mutation landscape analyses

Based on the distinction of patients in low- and high-risk groups, gene set enrichment analyses (GSEA) software (http://www.gsea-msigdb.org/gsea/login.jsp) was applied to discover the pathways that were mainly enriched in each group. The criterion of normal *p* < 0.05 and FDR < 0.25 was considered statistically significant. Gene Ontology (GO) and Kyoto Encyclopedia of Genes and Genomes (KEGG) pathway analyses were conducted using the “ClusterProfiler” R package, and FDR < 0.05 was considered statistically significant. The R package “maftools” was used to process somatic mutation data in each group. The first 30 mutated genes from the different groups were then shown.

### Estimation of the tumor immune microenvironment

The correlation between risk score and immune infiltrating level of immune cells was conducted using “scales”, “ggplot2”, “ggtext”, “tidyverse”, “ggpubr”, and “limma” R packages. During this process, the immune infiltration statuses in MIBC patients were obtained from XCELL, TIMER, QUANTISEQ, MCPcounter, EPIC, CIBERSORT-ABS, and CIBERSORT on TIMER 2.0 (http://timer.cistrome.org/). The corrected infiltration scores of related immune cells and pathways were calculated using the “GSVA” R package through the single-sample gene set enrichment analysis (ssGSEA). Meanwhile, the ESTIMATE score, including stromal score and immune score, was achieved using the “ESTIMATE” R package, which may represent the tumor immune infiltration level of each sample.

### The potential therapeutic value of the model

In order to evaluate the potential therapeutic value of this model in MIBC patients, we compared the half-maximal inhibitory concentration (IC50) of specified chemotherapy drugs that were applied to MIBC between low- and high-risk groups through the “pRRophetic” R package. The data on transcriptional expression and drug response were retrieved from the Genomics of Drug Sensitivity in Cancer database (GDSC, http://www.cancerrxgene.org/downloads).

## Results

### NLRs in muscle-invasive bladder cancer patients

**FIGURE 1:**
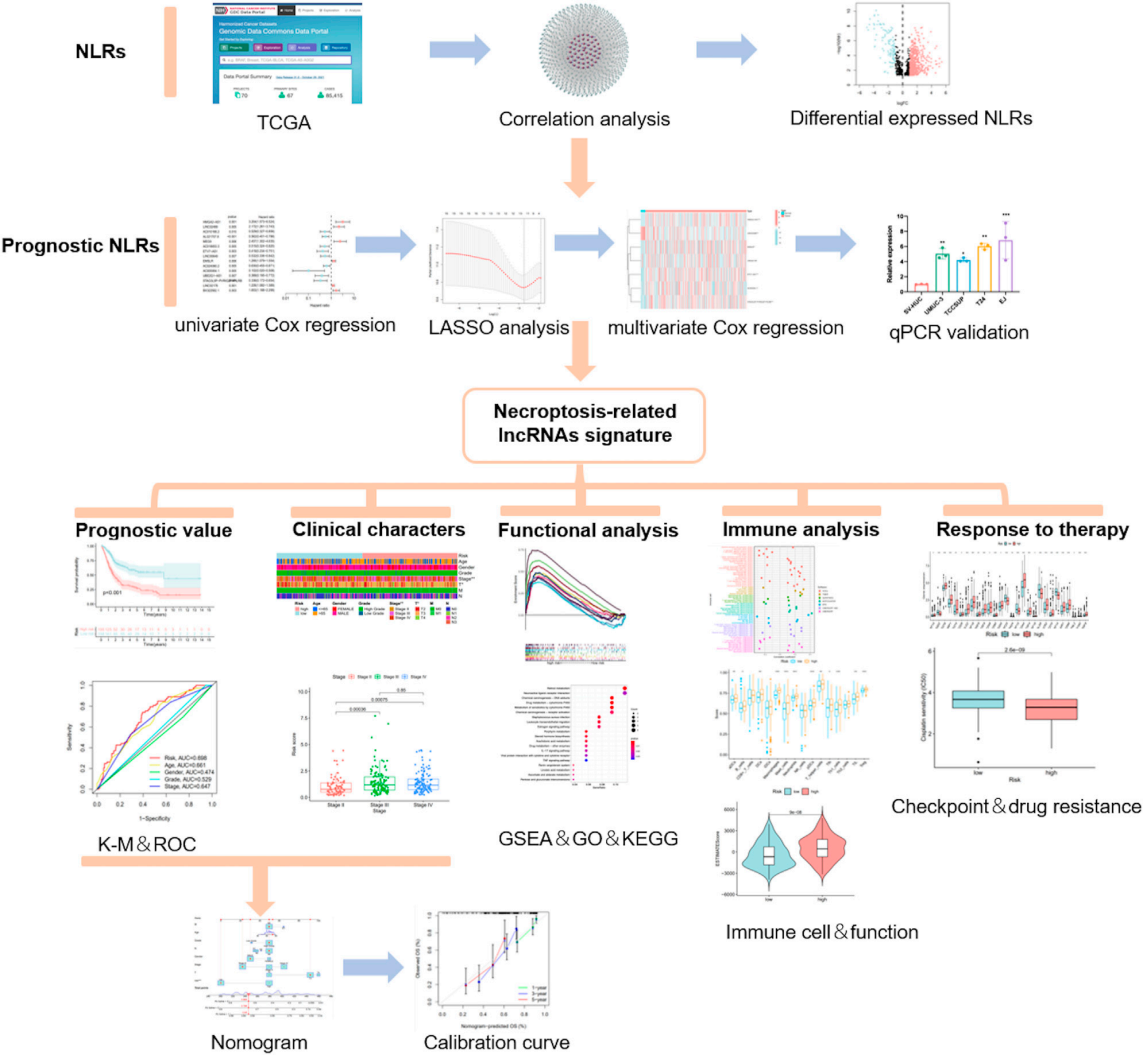
Flow chart.

**FIGURE 2:**
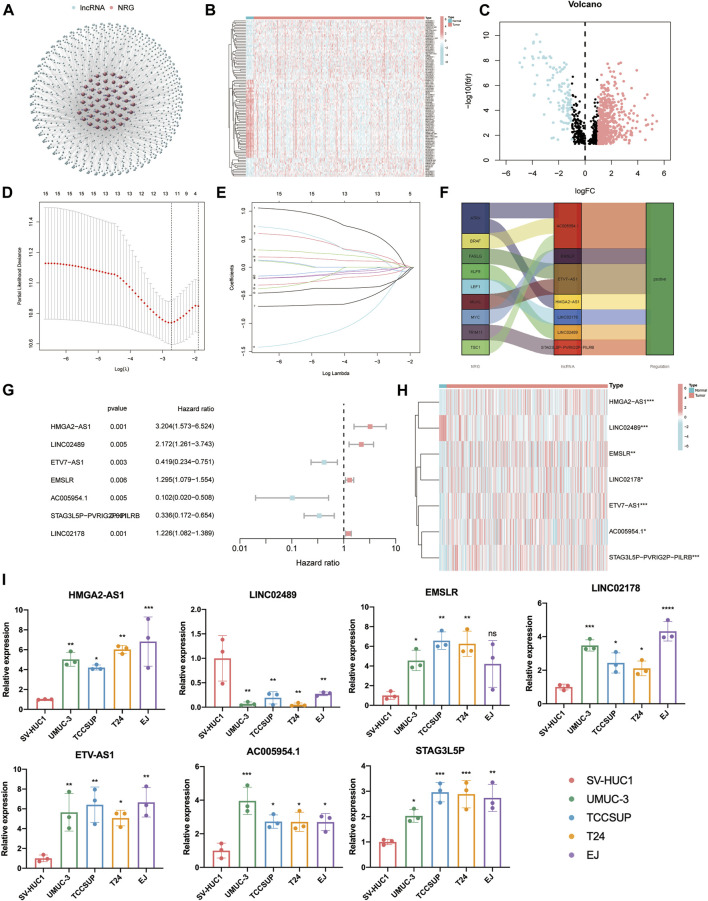
Identification of prognostic necroptosis-related lncRNAs (NLRs) in muscle-invasive bladder cancer (MIBC). **(A)** The network figure of necroptosis-related genes (NRGs) and NLRs (correlation coefficient > 0.4 and *p* < 0.001). **(B)** The heat map of different NLRs between tumor and normal samples. **(C)** The volcano plot of 689 differentially expressed NLRs. **(D)** The cross-validation for variable selection in the least absolute shrinkage and selection operator (LASSO) model. **(E)** The LASSO coefficient profile of 12 NLRs. **(F)** The Sankey diagram of the relationship between necroptosis-related genes (NRGs) and NLRs. **(G,H)** The forest plot and heat map of determined prognostic NLRs that executed by multivariate Cox proportional hazard regression analysis. **(I)** The qRT-PCR results of seven prognostic NLRs in bladder cancer cells. *p < 0.05, **p < 0.01, ***p < 0.001, ****p < 0.0001. ns, not significant.

The flow chart of this study is shown in Figure 1. By eliminating tumor samples with T0-1 grade, which were defined as NMIBC, a total of 407 MIBC and 19 normal samples were eventually considered in our research. We first evaluated the expression differences of NRGs between tumor and normal tissues (Supplementary Figure S1A). In addition, we also evaluated the mutation frequency of NRGs in MIBC, and the top 30 mutated genes are presented in Supplementary Figure S1B. Then, according to the correlation to NRGs, 1,139 lncRNAs were screened as NLRs. The figure of the network between NRGs and NLRs is exhibited in Figure2A (correlation coefficient > 0.4 and *p* < 0.001). Finally, 689 NLRs were found differentially expressed between tumor and normal samples (Figure 2B), of which 579 were upregulated and 110 were downregulated (Figure 2C).

### Identification of prognostic necroptosis-related differentially expressed lncRNAs

After univariate Cox proportional hazard regression analysis (*p* < 0.01), 15 lncRNAs were found to be associated with the prognosis of MIBC patients. The forest plot and heat map of these lncRNAs are displayed in Supplementary Figures S2A,B. Then, in order to overfit the prognostic signature, 12 lncRNAs were identified as candidate NLRs through the LASSO-penalized Cox analysis with minimum lambda value (Figures 2D,E). Eventually, we executed the stepwise multivariate Cox proportional hazard regression analysis (Figure 2G) to sort out seven defined NLRs (HMGA2-AS1, LINC02489, ETV7-AS1, EMSLR, AC005954.1, STAG3L5P-PVRIG2P-PILRB, and LINC02178) that can be identified as prognostic NLRs (Supplementary Table S2). The heat map exhibited the different expression of these seven NLRs between normal and tumor samples (Figure 2H). Meanwhile, we visualized the relationship between NRGs and these NLRs with the Sankey diagram (Figure 2F). To further confirm the differential expression in MIBC, qPCR analysis was carried out in four BLCA cells (UMUC-3, TCCSUP, T24, and EJ) and a normal bladder epithelial cell (SV-HUC1). It is evident that HMGA2-AS1, EMSLR, LINC02178, ETV7-AS1, AC005954.1, and STAG3L5P-PVRIG2P-PILRB were upregulated in BLCA cells, while LINC02489 was significantly lower in BLCA cells (Figure 2I).

### The molecular subtype based on prognostic NLRs

To identify the clinical value of these prognostic NLRs in the classification of MIBC patients, we divided all MIBC patients into several molecular subtypes by consensus clustering analysis based on the seven aforementioned NLRs. As a result, all MIBC patients were regrouped into two clusters (k = 2) on account of the highest intragroup relationships and the lowest intergroup relationships (Supplementary Figure S3A). The Kaplan–Meier curve showed that MIBC patients in cluster 1 had a notably improved survival rate than those in cluster 2 (*p* = 0.006, Supplementary Figure S3B). Previous studies suggested that necroptosis in cancer cells can mediate immune response by facilitating interaction between dying cancer cells and immune cells (Sprooten et al., 2020; Tang et al., 2020). Therefore, we compared the immune function of the two clusters to determine whether this classification has the predictive value of distinguishing immune response. Unfortunately, we found no difference in stromal score, immune score, and ESTIMATE score between the two clusters (Supplementary Figures S3C–E). In summary, we believe that this molecular subtype is not suitable for subsequent analysis.

### Construction and evaluation of prognostic value of necroptosis-related risk model

Due to the poor clinical value of classification into two clusters, we decided to construct a risk model same as previous studies (Chen et al., 2021c; Tang et al., 2021). According to the aforementioned prognostic NLRs and corresponding coefficient value, the risk score was calculated as follows: risk score = (0.9220 × HMGA2-AS1 expression) + (0.5187 × LINC02489 expression) + (−0.8027×ETV7-AS1 expression) + (0.2899 × EMSLR expression) + (−1.7005 × AC005954.1 expression) + (−0.5790 × STAG3L5P-PVRIG2P-PILRB expression) + (0.1412 × LINC02178 expression). Then, patients were categorized into two groups, termed as high-risk and low-risk, based on the median value of the risk score.

**FIGURE 3:**
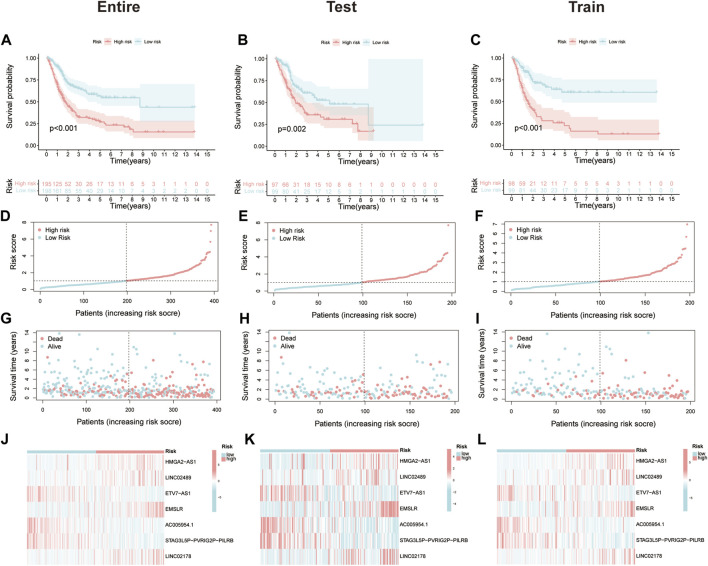
Prognostic value of the necroptosis-related risk model. **(A–C)** Kaplan–Meier survival curves of overall survival (OS) between high- and low-risk groups in the entire, test, and train cohort. **(D–F)** The distribution of risk score among muscle-invasive bladder cancer (MIBC) patients in the entire, test, and train cohort. **(G–I)** Exhibition of survival time and status between high- and low-risk groups in the entire, test, and train cohort. **(J–L)** The heat map of seven prognostic necroptosis-related lncRNAs (NLRs) expression in the entire, test, and train cohort.

In the beginning, PCA was used to display the distribution of patients in two clusters and risk groups (Supplementary Figure S4). After removing patients without prognostic information, all MIBC samples (*n* = 393) were randomly divided into test (*n* = 196) and train (*n* = 197), two cohorts to certify the prognostic value of this risk model. The characteristics of patients in the two cohorts are shown in Table 1. First, Kaplan–Meier analysis was used to analyze the OS of each group. Consistent with the results demonstrated in the entire cohort (*p* < 0.001, Figure 3A), it showed that the survival time of the low-risk cohort was significantly longer than that of the high-risk cohort in the test and train groups (*p* = 0.002, *p* < 0.001; Figures 3B,C). The risk scores of each group are exhibited in Figures 3D–F. Significantly, as the risk score increased, more and more patients died (Figures 3G–I). At last, the heat map visualized the expression of NLRs in each risk group. We found that the expressions of HMGA2-AS1, LINC02489, EMSLR, and LINC02178 were higher in the high-risk group, while ETV7-AS1, AC005954.1, and STAG3L5P-PVRIG2P-PILRB were expressed higher in the low-risk group (Figures 3J–L).

**TABLE 1:** Clinical characteristics of patients in different cohorts.

Variable	Entire (*n* = 393)	Test (*n* = 196)	Train (*n* = 197)
Age (years)			
≤65	158 (40.2%)	85 (43.4%)	73 (37.1%)
>65	235 (59.8%)	111 (56.6%)	124 (62.9%)
Gender			
Male	291 (74.0%)	146 (74.5%)	145 (73.6%)
Female	102 (26.0%)	50 (25.5%)	52 (26.4%)
Grade			
Low-grade	18 (4.6%)	9 (4.6%)	9 (4.6%)
High-grade	372 (94.7%)	187 (95.4%)	185 (93.9%)
Unknown	3 (0.7%)	0 (0%)	3 (1.5%)
Stage			
Stage II	125 (31.8%)	66 (33.7%)	59 (30.0%)
Stage III	136 (34.6%)	72 (36.7%)	64 (32.5%)
Stage IV	130 (33.1%)	58 (2.96%)	72 (36.5%)
Unknown	2 (0.5%)	0 (0%)	2 (1%)
T stage			
T2	117 (29.8%)	62 (31.6%)	55 (27.9%)
T3	188 (47.8%)	91 (46.4%)	97 (49.2%)
T4	57 (14.5%)	28 (14.3%)	29 (14.7%)
Tx + unknown	31 (7.9%)	15 (7.7%)	16 (8.1%)
N stage			
N0	227 (57.8%)	116 (59.2%)	111 (56.3%)
N1	44 (11.2%)	21 (10.7%)	23 (11.7%)
N2	75 (19.1%)	31 (15.8%)	44 (22.3%)
N3	7 (1.8%)	4 (2.0%)	3 (1.5%)
Nx + unknown	40 (10.2%)	24 (12.2%)	16 (8.1%)
M stage			
M0	187 (47.6%)	92 (46.9%)	95 (48.2%)
M1	10 (2.5%)	4 (2.0%)	6 (3.0%)
Mx + unknown	196 (49.9%)	100 (51.0%)	96 (48.7%)

**FIGURE 4:**
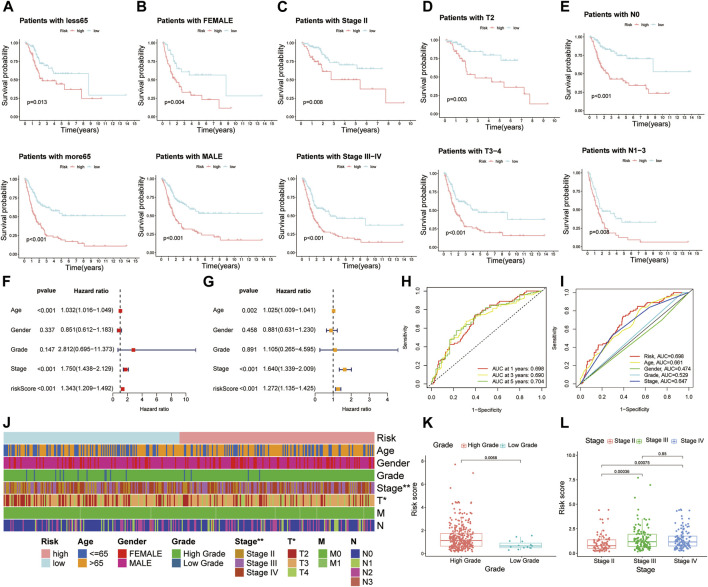
Further confirmation of prognostic value of the risk model combined with clinical features. Kaplan–Meier survival curves of high- and low-risk groups among muscle-invasive bladder cancer (MIBC) patients sorted based on different clinical features, including **(A)** age, **(B)** gender, **(C)** stage, **(D)** T stage, and **(E)** N stage. **(F,G)** Risk model was an independent factor of prognosis by using univariate Cox and multivariate Cox proportional hazard regression analyses. **(H)** The 1-, 2-, and 3-year receiver operating characteristics (ROC) curves of all MIBC patients. **(I)** The 1-year ROC curves of risk score and other clinical features. **(J)** The heat map of distinctions in clinical features between high- and low-risk groups. **(K,L)** The histogram showing the difference of risk scores in MIBC patients stratified by grade and stage. *p < 0.05, **p < 0.01.

Owing to the fact that patients in our study had different clinical features, we separated all patients into diverse groups to further proofread the prognostic value of this signature according to age, gender, stage, T stage, and N stage. As we expected, patients in the high-risk group showed worse OS than those in the low-risk group in each different classification, which was consistent with former analysis (Figures 4A–E).

To further identify whether the risk model was an independent factor of prognosis in MIBC patients, univariate Cox and multivariate Cox proportional hazard regression analyses were performed. As we can see, age (*p* < 0.001), stage (*p* < 0.001), and risk score (*p* < 0.001) were significantly associated with OS of MIBC patients through univariate Cox proportional hazard regression analysis (Figure 4F). Then, with the multivariate Cox proportional hazard regression analysis, age (*p* = 0.002), stage (*p* < 0.001), and risk score (*p* < 0.001) were defined as independent prognostic factors (Figure 4G).

The area under the ROC curve (AUC) was defined as the outcomes of ROC. The 1-, 3-, and 5-year AUC value of all MIBC patients were 0.698, 0.690, and 0.704, respectively (Figure 4H). In terms of the 1-year ROC of the risk model, it is evident that the AUC of the risk score was 0.698, which was better than other clinical variables in predicting the prognosis of MIBC patients (Figure 4I). The AUC values of 2 years and 3 years were also higher than those of other variables (Supplementary Figure S5). In general, all these indicated the remarkable ability of the risk model in predicting prognosis.

### Analysis of prognostic risk model and different clinical features

We presented the correlation between risk scores and clinical features from two aspects. On the one hand, the heat map displayed that stage and T stage of patients were different between high- and low-risk groups (Figure 4J). From another point of view, when MIBC patients were stratified by grade, stage, and T stage, a significant difference of risk score was observed from the histogram (Figures 4K,L; Supplementary Figure S6A). However, no distinction of risk score was observed in patients classified by age, gender, N stage, and M stage (Supplementary Figures S6B–E).

### Construction and calibration of nomogram

**FIGURE 5:**
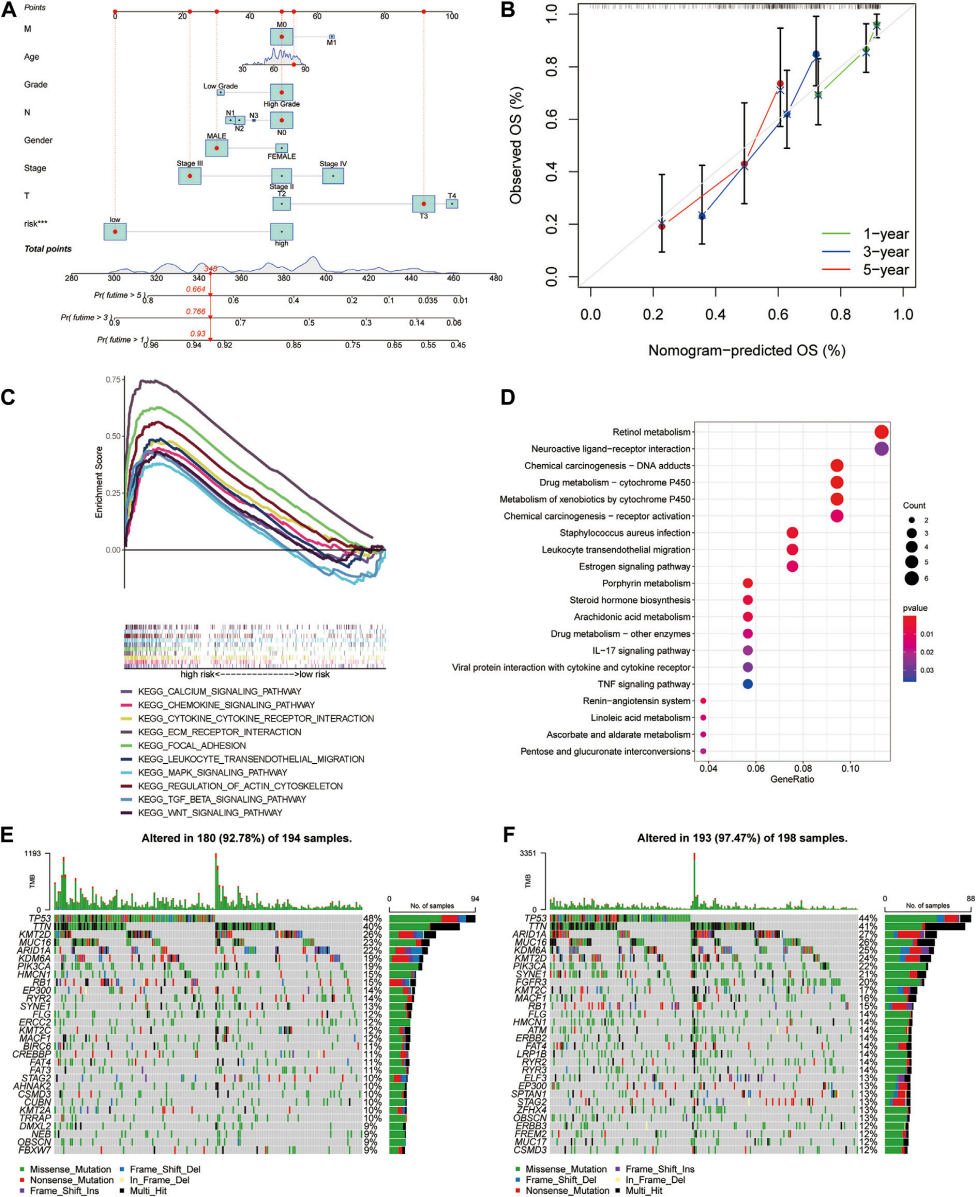
Construction and calibration of the nomogram, followed by functional and tumor burden analyses. **(A)** Nomogram integrated age, gender, grade, stage, T stage, N stage, M stage, and risk score. **(B)** Calibration curves test consistency of the nomogram at 1, 3, and 5 years. **(C)** Activated pathways analyzed by gene set enrichment analyses (GSEA) in the high-risk group. **(D)** Kyoto Encyclopedia of Genes and Genomes (KEGG) analysis of differential genes between high- and low-risk groups. **(E,F)** The gene mutations between high- and low-risk groups.

Referring to the findings mentioned earlier, in order to further predict the prognosis of MIBC patients, the nomogram was constructed as a consequential tool to predict 1-, 3-, and 5-year OS based on clinicopathological variables and risk model (Figure 5A). The calibration curve gave eloquent proof of consistency between the practical survival time and the predicted OS at 1, 3, and 5 years (Figure 5B). The C-index value of the risk model is 0.711.

### Functional analyses and tumor mutation landscape

To investigate the distinction of signaling pathways in different risk score groups, GSEA was performed. Patients in the low-risk group were mainly enriched in metabolism-related pathways (Supplementary Figure S7A). At the same time, the WNT signaling pathway, TGF-β signaling pathway, and MAPK signaling pathway were enriched in the high-risk group. In addition to these, the other pathways, including ECM receptor, focal adhesion, regulation of actin cytoskeleton, chemokine signaling pathways, and cytokine–cytokine receptor interaction, were highly concerned with immunity (Figure 5C). GO and KEGG analysis of different genes between high- and low-risk groups also showed that there are several immune-related pathways enriched (Supplementary Figure S7B; Figure 5D). Referring to the difference in cancer-related gene mutations between the high-risk and low-risk groups, we presented the most frequent somatic mutations in each group. The waterfall plot showed that the low-risk group acquired a higher gene mutation rate than the high-risk group (Figures 5E,F). We also calculated the tumor mutation burden (TMB) value for each patient and compared the difference between the high-risk and low-risk groups (*p* = 0.00031, Supplementary Figure S7C).

### Infiltration of related immune cells and pathways

**FIGURE 6:**
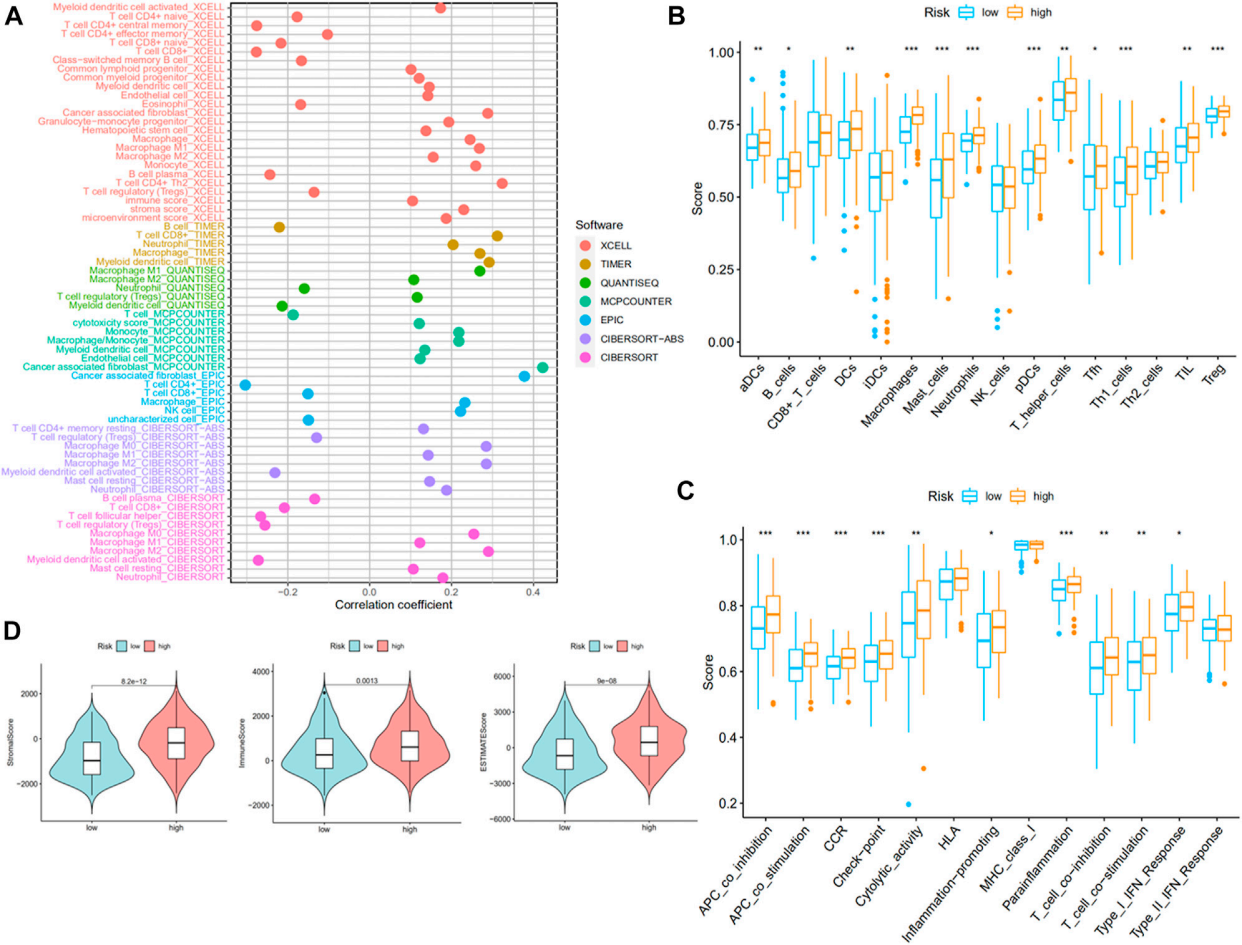
Immune infiltration of muscle-invasive bladder cancer (MIBC) patients between high- and low-risk groups. **(A)** The bubble chart presented a correlation between risk score and infiltrating level of immune cells. **(B,C)** The distinction of immune infiltrating cells and immune-related functions between low- and high-risk groups. **(D)** The stromal score, immune score, and ESTIMATE score of the two groups.

**FIGURE 7:**
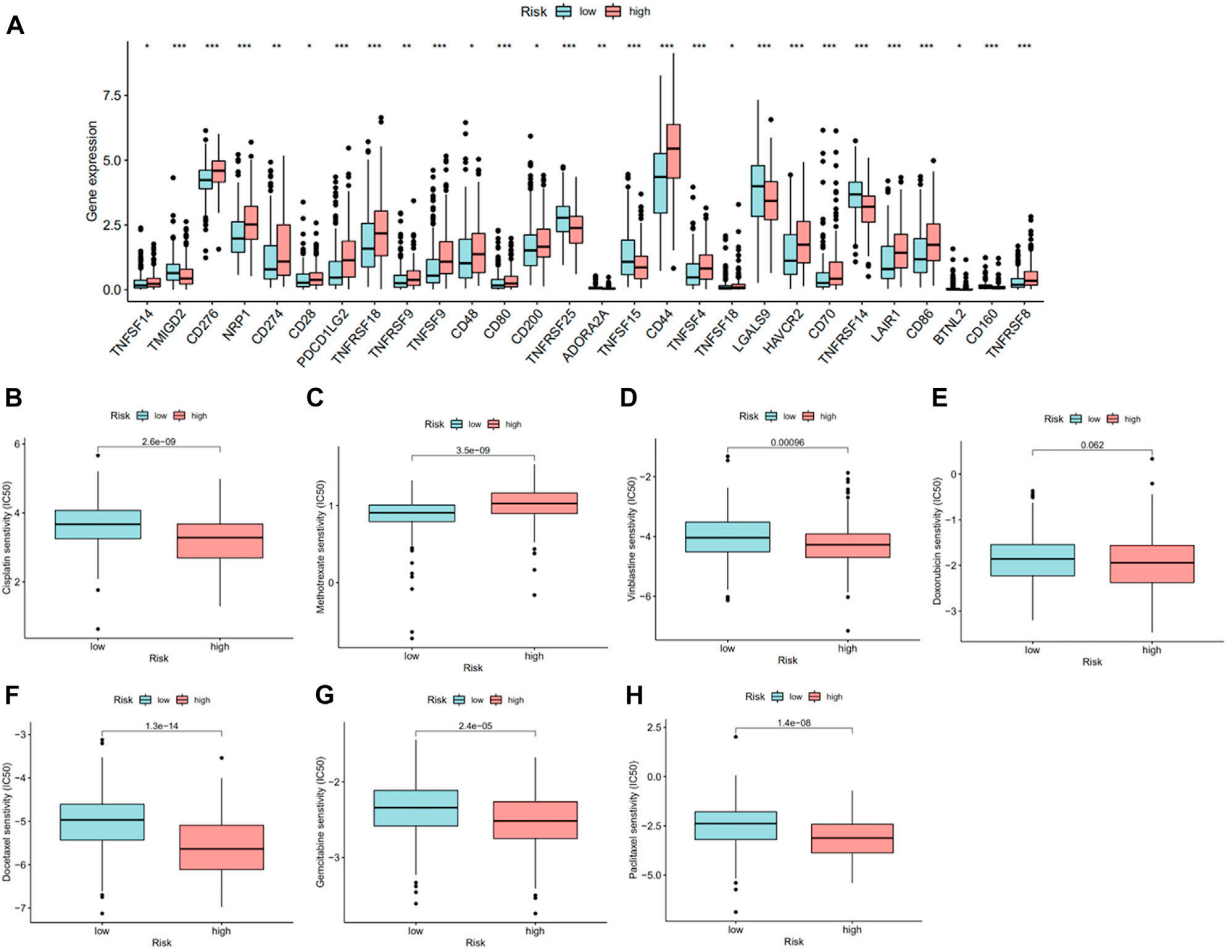
Checkpoint and drug response between high- and low-risk groups. **(A)** The significant distinction of checkpoint between high- and low-risk groups. **(B–H)** IC50 of cisplatin, methotrexate, vinblastine, doxorubicin, docetaxel, gemcitabine, and paclitaxel between high- and low-risk groups.

On account of immune-related pathways enriched in the high-risk group, we further explored the immune cells and functions in MIBC patients. First, we presented the correlation between risk score and infiltrating level of immune cells from different platforms at the bubble chart (Figure 6A). It is evident that the correlation coefficient of most immune cells was greater than 0. In other words, infiltrating levels of most immune cells such as macrophage, CD4+ T cell (Th2) at XCELL, T-cell CD8+, macrophage at TIMER, T-cell regulatory (Tregs) at QUANTISEQ, and NK cell at EPIC were positively correlated with the risk scores in MIBC patients. Then, the boxplot showed a significant distinction of immune cells and functions between low- and high-risk groups. In terms of immune-related cells (Figure 6B), MIBC patients in the high-risk group contained a higher percentage of activated dendritic cells (aDCs), B cells, dendritic cells (DCs), macrophages, mast cells, neutrophils, plasmacytoid dendritic cells (pDCs), T helper cells, T follicular helper (Tfh) cells, T helper type 1 (Th1) cells, tumor-infiltrating lymphocyte (TIL), and T regulatory cells (Tregs). Also, referring to immune functions (Figure 6C), there was significant distribution in antigen-presenting cell (APC) co-inhibition, APC co-stimulation, chemokine receptor (CCR), checkpoint, cytolytic activity, inflammation promotion, parainflammation, T-cell co-inhibition, T-cell co-stimulation, and type I IFN response between the two groups. Furthermore, MIBC patients with a higher risk score emerged with pronounced elevation of stromal score, immune score, and ESTIMATE score (Figure 6D). At last, we compared the immune checkpoint between the two groups and found that almost all checkpoints such as PD-L1 (CD274) were expressed higher in the high-risk group (Figure 7A). In summary, the aforementioned findings indicated that the high-risk group had a relative immune-activated state.

### Potential drug resistance in risk groups

As mentioned earlier, expression of PD-L1 was higher in the high-risk group, which suggested that patients in these two groups may show different sensitivities to immunotherapy, especially anti-PD-1/L1 immunotherapy. Chemotherapy is one of the most important treatments for MIBC patients in addition to immunotherapy. Therefore, for validating the potential therapeutic value of this risk model, we compared the IC50 of specified chemotherapy drugs between the two groups. We found that patients in the high-risk group were more sensitive to cisplatin, gemcitabine, vinblastine, paclitaxel, and docetaxel than those in the low-risk group, while patients in the low-risk group were more sensitive to methotrexate (Figures 7B–H).

## Discussion

Recently, it is widely accepted that nonapoptotic regulated cell death (RCD) is involved in the pathogenesis and therapeutic responses of various types of cancers (Stockwell et al., 2020; Hsu et al., 2021; Koren and Fuchs, 2021). Necroptosis is one of the nonapoptotic regulated cell death which differs from autophagy, ferroptosis, and pyroptosis. To our knowledge, there have been abundant studies that explored the role of autophagy, ferroptosis, pyroptosis, and other RCDs in MIBC (Chen et al., 2021b; Luo et al., 2021; Yan et al., 2021; Zhang et al., 2021). However, only a few studies have investigated the position of necroptosis in MIBC patients, and all of them focused on a single necroptosis-related gene (Yan et al., 2014; Nie et al., 2021). Our research is a comprehensive analysis of NLRs in MIBC, which can provide a reference for further studies to some extent.

In this study, we obtained seven prognostic NLRs from the TCGA database step by step, and a risk model was constructed based on the seven NLRs in MIBC patients. Then, according to the risk signature which was identified as an independent prognostic factor and other corresponding clinical features, a novel nomogram was established for predicting the prognosis for each patient. Specifically, we further verified that the risk model exhibits a splendid ability of predicting prognosis, immune microenvironment, and drug resistance.

To begin with, HMGA2-AS1, LINC02489, ETV7-AS1, EMSLR, AC005954.1, STAG3L5P-PVRIG2P-PILRB, and LINC02178 were sorted out as determined NLRs in MIBC patients for subsequent analyses. Among these selected NLRs, previous studies confirmed that some of them played crucial roles in cancers. For instance, a previous study discovered that HMGA2-AS1 was involved in positively mediating tumorigenesis of pancreatic cancer, and patients with a high level of HMGA2-AS1 may have relatively poor prognosis (Ros et al., 2019). Also, HMGA2-AS1 was found to be upregulated in osteosarcoma and laryngeal squamous cell carcinoma compared to their corresponding normal samples (Jing et al., 2020; Rothzerg et al., 2021). A study by Sun et al. (2020) showed that LINC02178 can predict the prognosis of BLCA patients, and Yan et al. (Li et al., 2018) confirmed its prognostic value for lung adenocarcinoma (LUAD) as well. With reference to EMSLR, it was proved to be essential for tumor-related phenotype. Cell-cycle phase distribution and proliferation reduction can be observed in lung cancer and colon cancer when depleting it (Hegre et al., 2021; Priyanka et al., 2022). Additionally, we estimated the expression of these several prognostic NLRs and found that it was basically consistent with the former bioinformatic analysis.

Instead of classifying all patients into two clusters, we establish a risk model based on seven prognostic NLRs like most studies. All MIBC patients were regrouped into high- and low-risk groups, and it is evident that patients in the low-risk group revealed a significant survival advantage. The first and foremost is to identify the prognostic value of the risk model. Not only did we demonstrate a significant difference in prognosis between the high- and low-risk groups, but this predictive signature can accurately predict prognosis without considering clinical features. We further recognized the risk model was an independent prognostic factor of OS by univariate Cox and multivariate Cox proportional hazard regression analyses. The AUC value of the risk model is the largest of all the variables we discussed, which means it is more effective than other current clinical features in predicting the OS of MIBC patients. Meanwhile, the relationship between clinical features and the risk model was explored in two ways. Thus, a nomogram integrating this risk model and other clinical characteristics was constructed for a more precise prediction of prognosis.

After evaluating the prognostic value of the risk model, GSEA was carried out to compare the signaling pathways between high- and low-risk groups. The results showed that the WNT signaling pathway, TGF-β signaling pathway, and MAPK signaling pathway were enriched in the high-risk group, which suggested that these NLRs may have an impact on the development and prognosis of MIBC through the aforementioned pathways. An increasing number of studies have proved the significance of these pathways in BLCA. For instance, lncRNA CASC9 can positively downregulate the expression of miR-497-5 as a microRNA sponge and subsequently activate the Wnt/β-catenin pathway, thus playing an oncogenic role in BLCA pathogenesis (Zhan et al., 2020). The TGF-β signaling pathway has been regarded as a potential mechanism for immunotherapy resistance resulting from its effects on TME in BLCA. It is exciting that several clinical studies combining immunotherapy with inhibitors of the TGF-β signaling pathway have achieved promising results in BLCA (Benjamin and Lyou, 2021). In addition, a previous study identified MAPK signaling as the core signaling pathway in MIBC (Schulz et al., 2021). The higher the MAPK activity in BLCA, the more malignant the traits of tumor progression, including tumor cell stemness, invasion, and epithelial–mesenchymal transition (EMT) (Kumar et al., 2009). Apart from these three signaling pathways, most other involved pathways were related to immunity. We speculate that the lncRNAs discussed in our study were related to necroptosis, and a series of emerging discovered evidence proved that necroptosis in cancer cells can be immunogenic. Not only can it interact directly with immune cells, but it can also initiate adaptive immune responses by releasing damage-associated molecular patterns (DAMPs), cytokines, and chemokines into the tumor microenvironment (Sprooten et al., 2020). The results of GO and KEGG analyses were consistent with the pathways during the process of necroptosis. However, the role of necroptosis in the induction and amplification of cancer immunity is complicated (Philipp et al., 2016; Gong et al., 2019). Combined with the key role of immunotherapy in the clinical treatment of MIBC, we further made an immunity analysis of the risk model.

Referring to immune cells, the results indicated that there was a positive correlation between risk score and infiltrating levels of most immune cells. Also, diverse risk groups were associated with significantly different levels of immune cell infiltration. Nowadays, it is widely believed that CD8+ T cells are the main focus in antitumor immunity. However, a groundbreaking study found that CD4+ T cell is the key point that influences the efficacy of immunotherapy in BLCA instead of CD8+ T cells (Oh et al., 2020). In our research, there was no difference in CD8+ T cells between the high- and low-risk groups, which is consistent with the study. At the same time, patients in the high-risk group had a higher abundance of CD4+ T cells, including helper T cells, Th1 cells, Tfh, and Tregs. Some researchers suggested that CD4+ T cells can predict clinical response to anti-PD-L1 (Oh et al., 2020) and are involved with better prognosis (Ahlen Bergman et al., 2018), but others found that CD4+ T cells can promote cancer metastasis in BLCA (Tao et al., 2018). Treg has been considered a suppressor of antitumor immunity for a long time (Tanaka and Sakaguchi, 2017). Interestingly, a study found that it has a positive effect on BLCA. Our study indicated that patients in the high-risk group have a higher infiltrating level of Tregs. Tumor-associated macrophage (TAMs) was another crucial factor in cancer growth, metastasis, and resistance to immunotherapy. In MIBC, a higher percentage of galectin 9-positive (Gal-9+) TAMs, a subtype of macrophages, was related to poorer prognosis accompanied by higher tumor stage and grade (Qi et al., 2019). Although there are no specific subtypes of macrophages in our study, the MIBC patients in the high-risk group showed higher infiltrating levels of macrophages with poorer outcomes and higher stages. Other immune cells, including mast cells, B cells, and neutrophils, were investigated to check whether they affected the balance between antitumor immunity and immune evasion in MIBC as well (Zhou et al., 2017; Liu et al., 2018; Jiang et al., 2019). In addition to the immune cells involved in our study, the immune responses were also compared between high- and low-risk groups. Multiple types of evidence identified the important role of necroptosis in the induction and amplification of cancer immunity. It has been found that RIPK3 was involved in the regulation of cytokine expression in DCs, which regulate immune homeostasis through modulating cytokines (Moriwaki et al., 2014). Some research studies also indicated that immunotherapeutic treatment should be customized according to the RIPK3 level (Gong et al., 2019). The significant immune responses between high- and low-risk groups demonstrate this point to a certain extent. Compared with the low-risk group, the expression of NLRs in the high-risk group was higher, thus inducing a stronger immune response. The estimate analysis further suggested that the patients in the high-risk group were more likely to benefit from immunotherapy. Altogether, this model may provide insights into individualized therapies by determining the response to immunotherapy.

Immune checkpoint was the key molecular target of immunotherapy, and recently, several immune checkpoint inhibitors have been approved for the treatment of MIBC. We found that the expression of almost all distinctive immune checkpoints was higher in the high-risk group. Nowadays, all approved immune checkpoint inhibitors for MIBC are PD1/PD-L1 inhibitors. Specifically, the expression of PD-L1 in the high-risk group was higher than that in the low-risk group, which was consistent with the higher abundance of immune cells in the high-risk group. Apart from immunotherapy, cisplatin-based chemotherapy remains the standard therapy of MIBC. Drug resistance is a critical reason for treatment failure and cancer-related death. Our research showed that patients in the high-risk group were sensitive to conventional chemotherapy drugs, including cisplatin, gemcitabine, vinblastine, paclitaxel, and docetaxel. All these indicated that patients in the high-risk group were more sensitive to immunotherapy, especially anti-PD1/PD-L1 immunotherapy and conventional chemotherapy. Taken together, this model may have important implications for the clinical translation of drug candidates, allowing adequate treatment in each case.

However, the study still had some limitations. First, the data used for constructing and validating the prognostic signature in our study were from a single source: TCGA. We did not use other data, such as the GEO database, for external verification to make the risk model more reliable. Second, the mechanism of involved NLRs in MIBC should be further discussed.

In conclusion, necroptosis is closely related to the development of MIBC. The novel risk signature based on seven significant NLRs act as an invaluable tool in predicting prognosis, immune microenvironment, and drug resistance, which may offer a basis for future studies.

## Data Availability

The original contributions presented in the study are included in the article/Supplementary Material; further inquiries can be directed to the corresponding author.

## Supplementary material

SUPPLEMENTARY FIGURE S1 Expression and gene mutation of necroptosis-related genes (NRGs). **(A)** The expression differences of NRGs between tumor and normal tissues. **(B)** The gene mutations of NRGs in muscle-invasive bladder cancer.

SUPPLEMENTARY FIGURE S2 Identification of prognostic necroptosis-related lncRNAs (NLRs) by univariate Cox regression analysis. **(A)** The forest plot of prognostic NLRs acquired through univariate Cox regression analysis. **(B)** The heat map of prognostic NLRs extracted by univariate Cox regression analysis.

SUPPLEMENTARY FIGURE S3 Survival and immune functions of two clusters based on prognostic NLRs. **(A)** MIBC patients were divided into two clusters by consensus clustering analysis. **(B)** Kaplan–Meier curve of OS in clusters. **(C–E)** The comparison of stromal score, immune score, and ESTIMATE score in two clusters.

SUPPLEMENTARY FIGURE S4 PCA analysis of patients in two clusters and risk groups. **(A)** Cluster. **(B)** Risk.

SUPPLEMENTARY FIGURE S5 ROC curves of risk score and other clinical features. **(A)** 2 years. **(B)** 3 years.

SUPPLEMENTARY FIGURE S6 Histogram showing the difference of risk scores in muscle-invasive bladder cancer (MIBC) patients stratified by clinical features. **(A)** T stage. **(B)** N stage. **(C)** M stage. **(D)** Age. **(E)** Gender.

SUPPLEMENTARY FIGURE S7 Functional analyses and tumor mutation burden (TMB). **(A)** Activated pathways analyzed by gene set enrichment analyses (GSEA) in the low-risk group. **(B)** Gene Ontology (GO) analysis of differential genes between high- and low-risk groups. **(C)** The differences in TMB between high- and low-risk groups.
