# Supplementary figures and images for: Prognostic implications of necroptosis-related long noncoding RNA signatures in muscle-invasive bladder cancer
Source: Front Genet. 2022 Dec 2;13:1036098. doi: 10.3389/fgene.2022.1036098 (PMC9755502; doi:10.3389/fgene.2022.1036098)

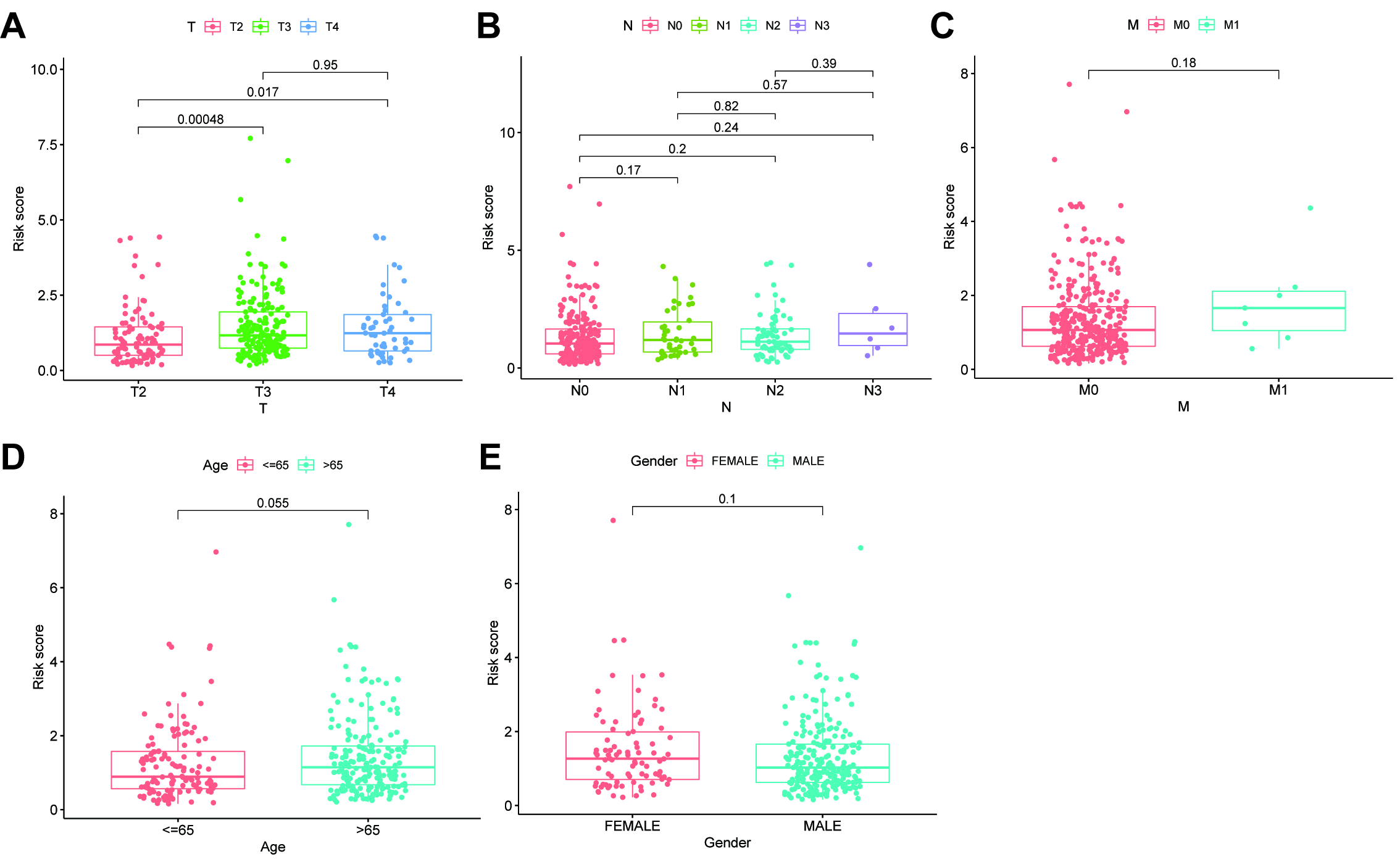

Supplement: Supplementary file 1 [file Image6.TIF]

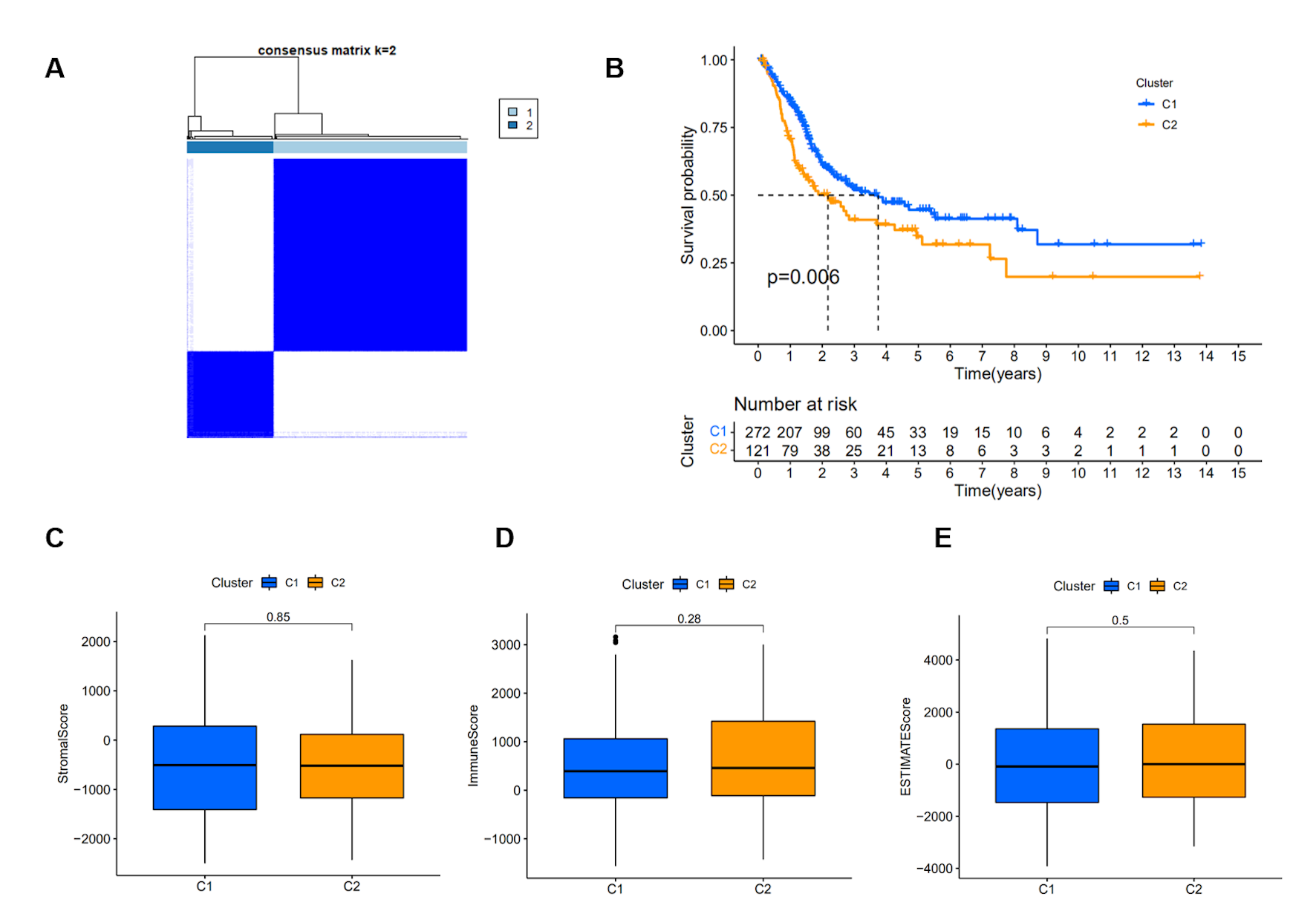

Supplement: Supplementary file 3 [file Image3.TIF]

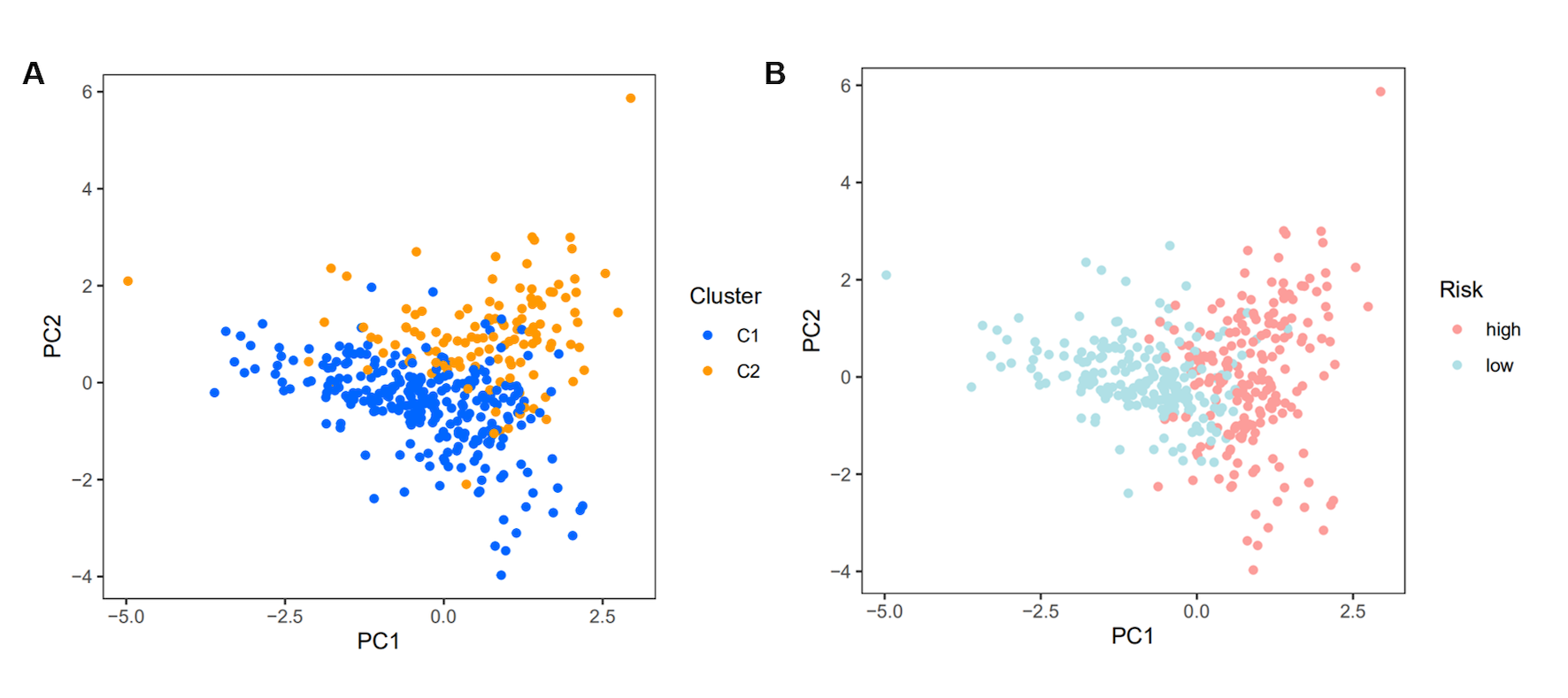

Supplement: Supplementary file 4 [file Image4.TIF]

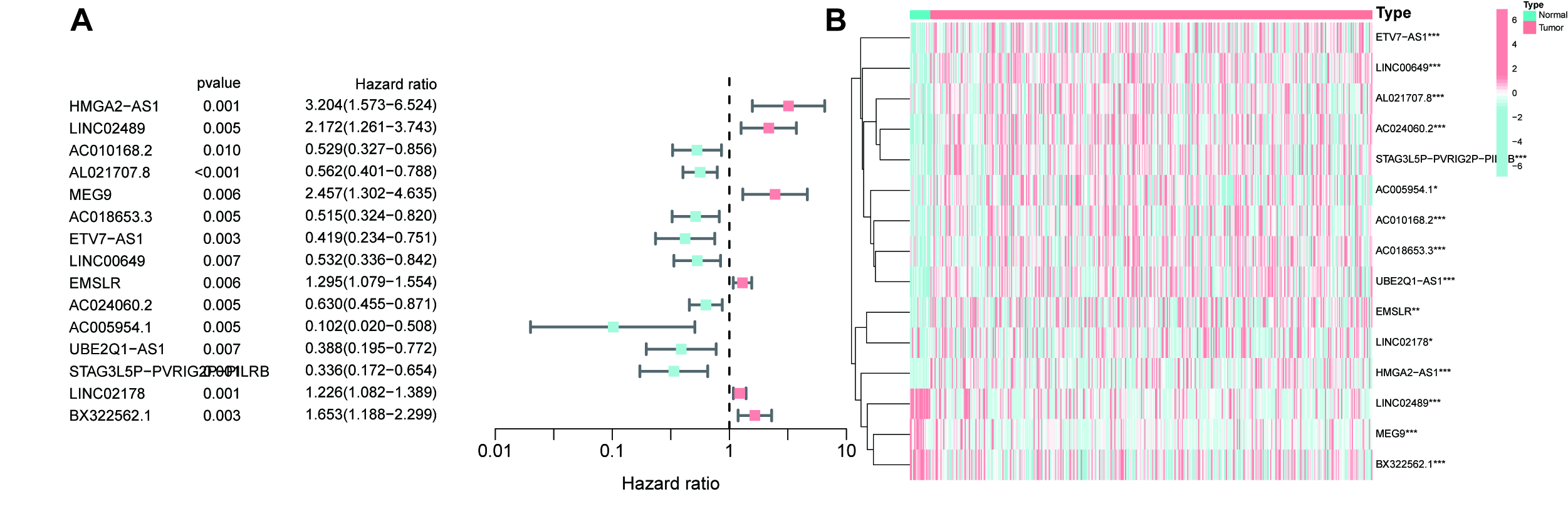

Supplement: Supplementary file 5 [file Image2.TIF]

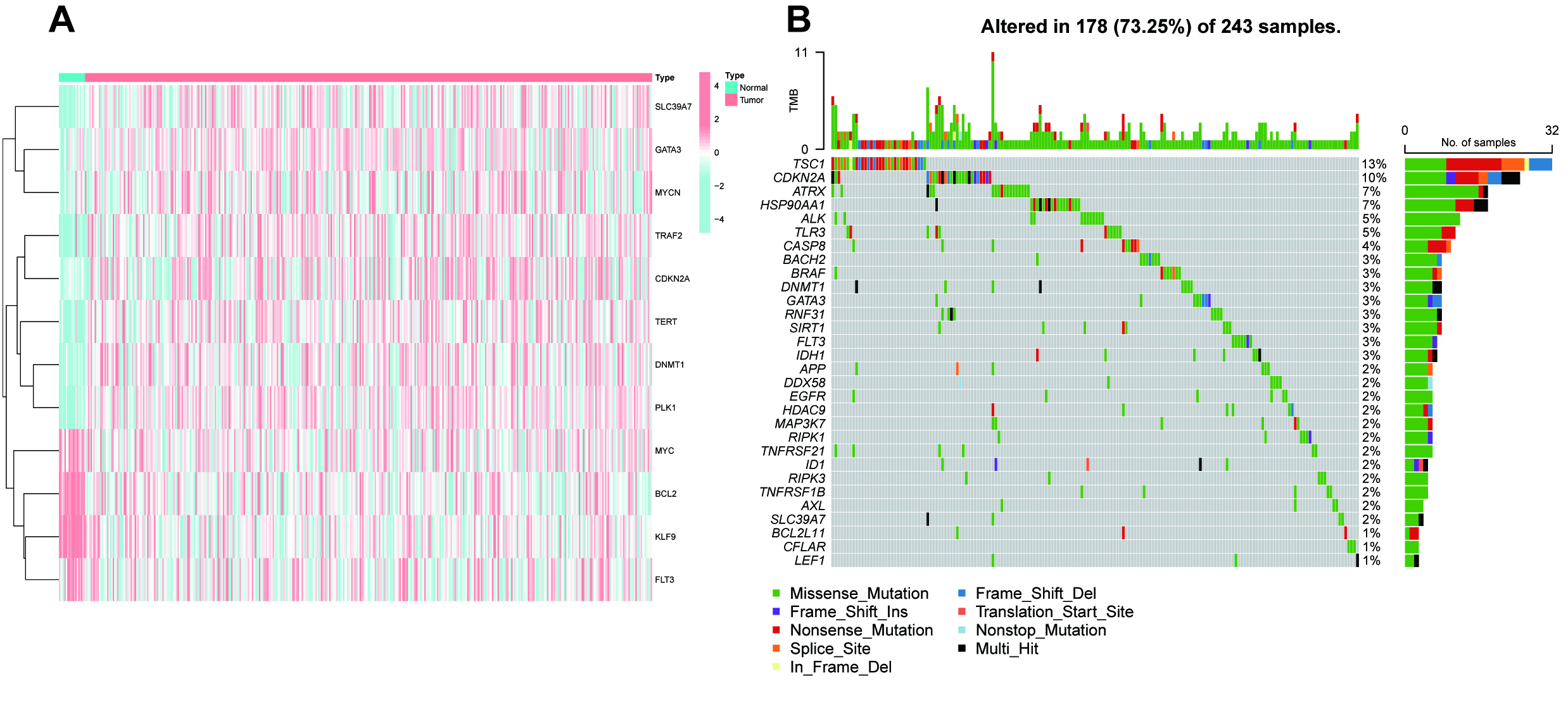

Supplement: Supplementary file 6 [file Image1.TIF]

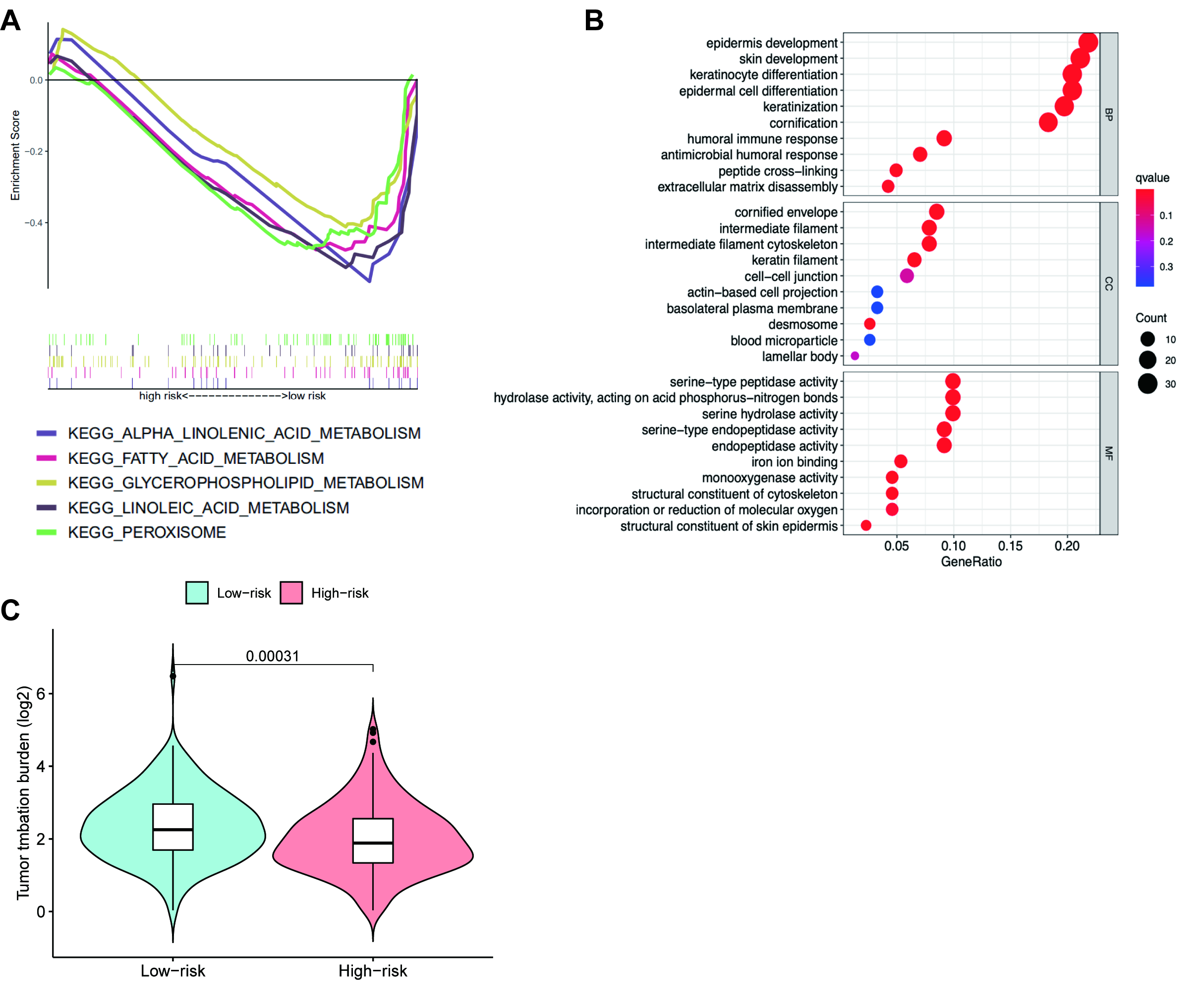

Supplement: Supplementary file 7 [file Image7.TIF]

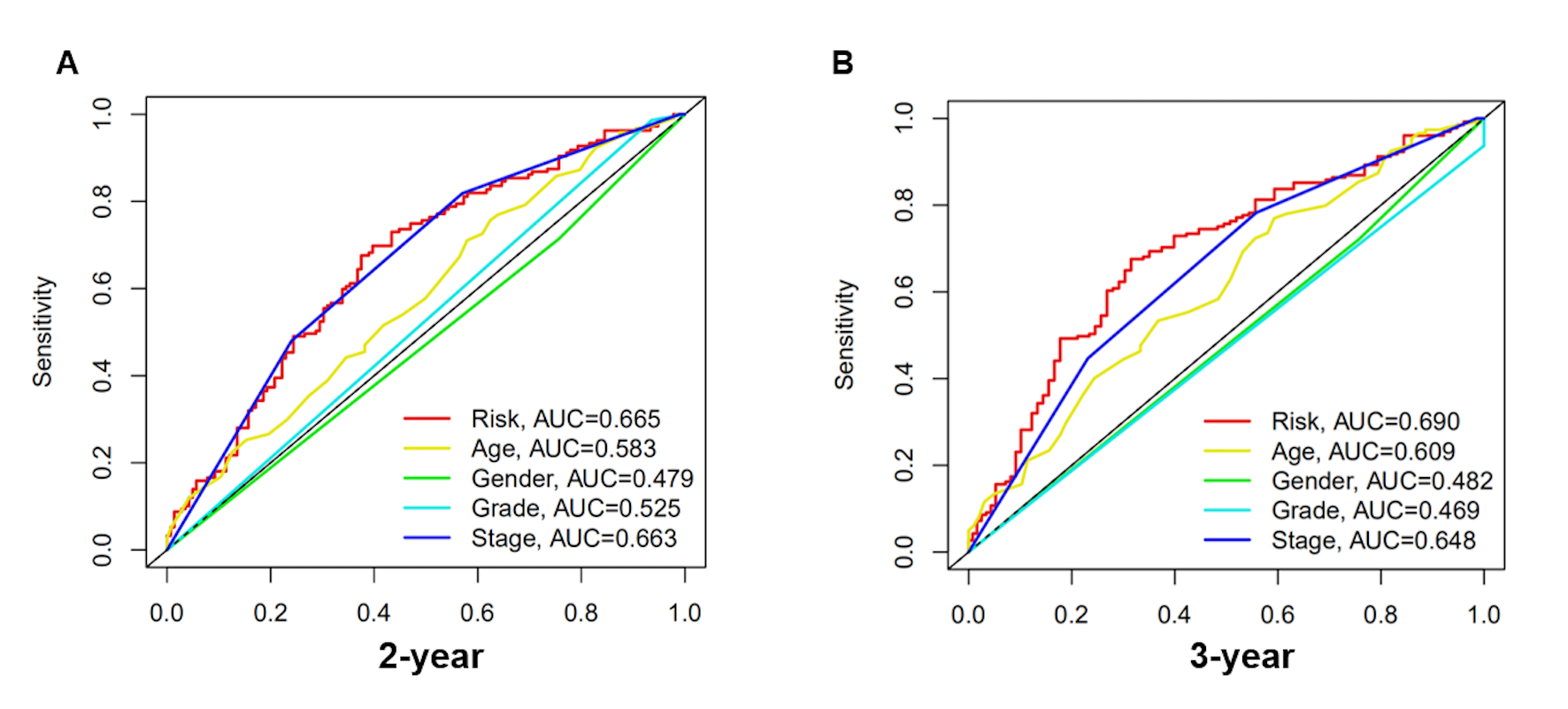

Supplement: Supplementary file 9 [file Image5.TIF]
