# Supplementary material for: Prognostic implications of necroptosis-related long noncoding RNA signatures in muscle-invasive bladder cancer
Source: Front Genet. 2022 Dec 2;13:1036098. doi: 10.3389/fgene.2022.1036098 (PMC9755502; doi:10.3389/fgene.2022.1036098)
Supplement: Supplementary file 2 [file Table1.DOCX]

**Supplementary Table1** |Oligonucleotide sequences used in the present study

| Primers | Sequences (5’-3’) | |
| --- | --- | --- |
| HMGA2-AS1 | Forward | TTCAAGCCATTCTCCTGCCTCAG |
|  | Reverse | GAAAACCTCACCAATCCCCA |
| LINC02489 | Forward | CTGCAAACACCACCTCCACA |
|  | Reverse | TGCCCTCCATCTTCCACACA |
| ETV7-AS1 | Forward | AGAAGGGGCTCAAGGCACAG |
|  | Reverse | AGGTGTTGCTGCATTTTTTCTGG |
| EMSLR | Forward | TGACGGGAACCGAAGGAGC |
|  | Reverse | TGGGCGACAGAGCGAGAC |
| AC005954.1 | Forward | CACTCACCAAGTCTCACCCC |
|  | Reverse | TGGACACAGCCATCTGCTCT |
| STAG3L5P-PVRIG2P-PILRB | Forward | AGGAAAGGAGGATAGTTGTTGG |
|  | Reverse | TCTGGGTAGCTCTGGAGGGTAC |
| LINC02178 | Forward | TGAGGGTGATGTCAACACAACAGC |
|  | Reverse | GGGGGCACAGAGAGAACTGG |
|  | Reverse | TAGGTGTCAAGGACTCTGC |
| β-actin | Forward | ATGACTTAGTTGCGTTACACC |
|  | Reverse | GACTTCCTGTAACAACGCATC |
