# Supplementary material for: Prognostic implications of necroptosis-related long noncoding RNA signatures in muscle-invasive bladder cancer
Source: Front Genet. 2022 Dec 2;13:1036098. doi: 10.3389/fgene.2022.1036098 (PMC9755502; doi:10.3389/fgene.2022.1036098)
Supplement: Supplementary file 8 [file Table2.DOCX]

**Supplementary Table 2** The detailed information of 7 prognostic NLRs

| Gene | Normal Mean | Tumor Mean | Log FC | P Value | Fdr |
| --- | --- | --- | --- | --- | --- |
| HMGA2-AS1 | 0.012521 | 0.117823 | 3.234193 | 0.000375 | 0.001057 |
| LINC02489 | 1.3934527 | 0.167619 | -3.05541 | 8.78E-11 | 6.38E-09 |
| ETV7-AS1 | 0.1823921 | 0.541057 | 1.568737 | 0.000153 | 0.000513 |
| EMSLR | 0.4636522 | 2.419548 | 2.383623 | 0.002534 | 0.004633 |
| AC005954.1 | 0.057659 | 0.145333 | 1.333746 | 0.010528 | 0.014417 |
| STAG3L5P-PVRIG2P-PILRB | 0.4891221 | 0.99704 | 1.027457 | 1.32E-05 | 6.84E-05 |
| LINC02178 | 0.886027 | 2.731846 | 1.624453 | 0.012885 | 0.017044 |
